# Composition of Conditioned Media from Radioresistant and Chemoresistant Cancer Cells Reveals miRNA and Other Secretory Factors Implicated in the Development of Resistance

**DOI:** 10.3390/ijms242216498

**Published:** 2023-11-19

**Authors:** Daria Molodtsova, Denis V. Guryev, Andreyan N. Osipov

**Affiliations:** 1 N.N. Semenov Federal Research Center for Chemical Physics, Russian Academy of Sciences, 119991 Moscow, Russia; dmolodtsova@gmail.com; 2 State Research Center—Burnasyan Federal Medical Biophysical Center of Federal Medical Biological Agency (SRC—FMBC), 123098 Moscow, Russia; denis.guryev@gmail.com; 3 Joint Institute for Nuclear Research, 6 Joliot-Curie St., 141980 Dubna, Russia

**Keywords:** radioresistance, chemoresistance, cancer cell, conditioned media, secretory factors, oncosomes, exosomes, miRNA

## Abstract

Resistance to chemo- or radiotherapy is the main obstacle to consistent treatment outcomes in oncology patients. A deeper understanding of the mechanisms driving the development of resistance is required. This review focuses on secretory factors derived from chemo- and radioresistant cancer cells, cancer-associated fibroblasts (CAFs), mesenchymal stem cells (MSCs), and cancer stem cells (CSCs) that mediate the development of resistance in unexposed cells. The first line of evidence considers the experiments with conditioned media (CM) from chemo- and radioresistant cells, CAFs, MSCs, and CSCs that elevate resistance upon the ionizing radiation or anti-cancer drug exposure of previously untreated cells. The composition of CM revealed factors such as circular RNAs; interleukins; plasminogen activator inhibitor; and oncosome-shuttled lncRNAs, mRNAs, and miRNAs that aid in cellular communication and transmit signals inducing the chemo- and radioresistance of sensitive cancer cells. Data, demonstrating that radioresistant cancer cells become resistant to anti-neoplastic drug exposure and vice versa, are also discussed. The mechanisms driving the development of cross-resistance between chemotherapy and radiotherapy are highlighted. The secretion of resistance-mediating factors to intercellular fluid and blood brings attention to its diagnostic potential. Highly stable serum miRNA candidates were proposed by several studies as prognostic markers of radioresistance; however, clinical studies are needed to validate their utility. The ability to predict a treatment response with the help of the miRNA resistance status database will help with the selection of an effective therapeutic strategy. The possibility of miRNA-based therapy is currently being investigated with ongoing clinical studies, and such approaches can be used to alleviate resistance in oncology patients.

## 1. Introduction

Chemoradiation therapy (CRT) is a commonly indicated treatment in the case of cancer. In combination with surgery or alone, it offers a relief of condition or even a cure for some patients. In cases when cancer cannot be abolished with CRT, resistance is often the case. Resistance can be a pre-existing tumor state due to a genetic profile or presence of cancer stem cells (CSCs), cancer-associated fibroblasts (CAFs), or mesenchymal stem cells (MSCs), or it can be acquired. Previous treatment with radiation or chemotherapy (CT) can lead to the selection of surviving cells with a resistant phenotype and/or the transformation of surviving cancer cells into resistant cells. This resistant phenotype allows cancer cells to escape death after future treatment and maintain a viable tumor cell population. Various mechanisms have been described that are associated with resistance, and many are shared between radioresistant and chemoresistant cells. Increased repair capacity, abrogation of cell cycle arrest, apoptosis evasion, tumor heterogeneity, activation of CSCs, mutation, and tumor microenvironment (TME) are common mechanisms leading to both radio- and chemoresistance. When anti-cancer therapy is administered, tumor cells exert a complex response that includes a state-specific secretory profile that orchestrates communication between cells to help them adapt. A variety of molecules, including cytokines, interleukins, mRNA, and ncRNA, such as miRNA and lncRNA, as well as others, are released into the extracellular space and can cause changes in the cells that uptake them and also reflect the cell-specific state. In this article, we describe how resistance-associated cancer secretome exerts its biological functions and its applications in diagnostics. We also highlight different therapeutic approaches to sensitize resistant cells and CSCs to achieve consistent clinical outcomes.

## 2. Conditioned Media

To reveal the influence of TME on the development of resistance of cancer cells, experiments with conditioned media (CM) were conducted. CM contains various paracrine factors and oncosomes that convey molecular signals and aid in cellular communication. Among the resistance-associated secretory molecules, circular RNAs (circRNA) and cytokines have been identified. Cytokines bind to cellular receptors and initiate a signal cascade, while circRNA can enter by endocytosis. circITGB6 was found to be associated with cisplatin resistance in M2 macrophages, while circATP8B4 facilitates higher viability after ionizing radiation (IR) treatment [1,2]. In addition to circRNA, interleukin-11 (IL-11), secreted by cancer-associated fibroblasts, previously treated with cisplatin, also induced a significantly higher viability of A549 cells following cisplatin exposure by activating the IL-11R/STAT3 anti-apoptotic signaling pathway [3].

Other studies explored radioresistance induction and showed that CM from previously irradiated cells also showed protective effects in the A549 cells upon additional X-ray exposure, leading to lower apoptotic rates [4]. CM from three types of previously irradiated lung cancer cells facilitated decreased cell death upon irradiation of sensitive cells via increased plasminogen activator inhibitor-1 (PAI-1) that upregulated AKT and ERK1/2 pathways and inhibited caspase-3 activity [5].

In vivo TME contains senescent cells that are formed after anti-cancer therapy that express the senescence-associated secretory phenotype (SASP). Senescent cells are more resistant to exposure due to their dormant state, polyploidy, and apoptosis evasion via senescent cell anti-apoptotic pathways, and they secrete various cytokines such as TGFβ1, TGFβ3, IL1β, IL-6, IL-8, CXCL1, CXCL2, and CXCL5 in addition to miRNA containing oncosomes that can promote tumor progression and confer subsequent therapy resistance [6,7]. Therapeutic approaches help to ablate senescent cells and were shown to improve treatment outcomes, as discussed in Section 3.1.

### 2.1. Oncosomes

Recently, oncosomes have attracted attention after studies highlighted these tiny vesicles as cargo for representative molecules from their donor cells and their role in the horizontal transfer of these molecules, such as mRNA, lncRNA, miRNA, proteins, lipids, and others. Oncosomes are exosomes released from cancer cells carrying cargo that promotes carcinogenesis such as oncogenes and other molecules facilitating tumor progression [8]. Research shows that chemoresistant cells offer protective effects to non-resistant cancer cells via the transfer of oncosomes. Multiple experiments demonstrated that incubation of sensitive cancer cells with oncosomes, isolated from the conditioned media of resistant cells, led to better viability after CT drug treatment [9,10,11,12]. Upon blocking oncosome release in combination with chemotherapy administration, a sensitizing effect was observed with higher treatment efficiency in different types of cancer cells, and it was also confirmed in vivo with a chorio-allantoic membrane (CAM) assay [10,13]. A CAM assay involves the deposition and growth of a tumor or other patient cells on top of the extraembryonic membrane of the developing chick embryo. This approach is valued as a model to recreate a tumor microenvironment due to the presence of immune cells, angiogenesis, and the extracellular matrix. In addition to shuttling molecules, oncosomes can directly facilitate chemoresistance, as was shown in case of breast cancer oncosomes that can reduce trastuzumab bioavailability by binding it with the human epidermal growth factor receptor-2 ligands that are present on their surface [9]. Oncosomes isolated from chemoresistant osteosarcoma and prostate cancer cells carried the MDR-1/P-glycoprotein itself and its mRNA and were associated with higher drug resistance of sensitive cells due the upregulation of this drug efflux pump [14,15]. Oncosome-shuttled mRNA of O6-alkylguanine DNA alkyltransferase and lncRNA that increases the expression of XRCC4 were shown to enhance DNA damage repair, thus mediating resistance to temozolomide in some cancer cells [16,17].

**Figure 1:**
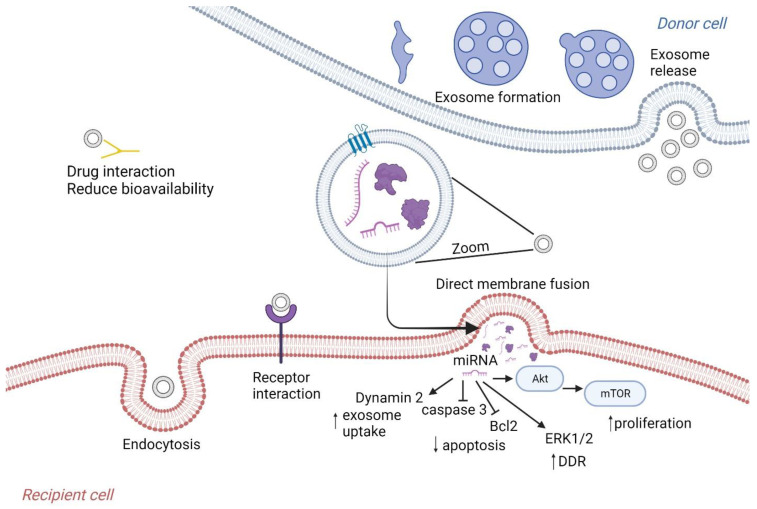
Role of exosomes in development of chemo- and radioresistance in sensitive cancer cells. Abbreviations: DDR—DNA damage response.

Research of oncosomes in irradiated cells showed that oncosome biogenesis and secretion appears to increase following irradiation, mediated by DNA-damage-induced p53 upregulation [18,19,20,21,22,23,24], and such an increase in oncosome secretion was also described after chemotherapy drug exposure [25,26]. Incubation of squamous cell carcinoma cells with oncosomes, isolated from previously irradiated cells, provided protective effects by increasing sensitive cell survival and decreasing the number of DNA double strand breaks (DSBs) after 6 h following radiation exposure [22]. Treatment of different lines of glioblastoma and breast cancer cells with oncosomes derived from previously irradiated cells also increased their survival after irradiation [23,24]. The role of oncosomes in the development of chemo- and radioresistance is depicted in Figure 1.

### 2.2. miRNA

There is growing evidence that resistance can develop via the delivery of oncosome-shuttled miRNA [9,10,27,28,29]. MicroRNA is a non-coding RNA molecule that is involved in the post-transcriptional regulation of gene expression by binding to mRNA and silencing genes, as well as under some circumstances, such as starvation, activating genes [30,31,32]. Extracellular miRNA acts in cellular communication, as it passes the signal from one cell to another via oncosome fusion with the recipient cell or by binding to Toll-like receptors and activating an intracellular signal cascade [30,33]. miRNA is involved with many important biological processes, including the development and disease, and some of them are evolutionarily conserved between species [34,35].

Numerous studies conducted microarray analyses and RT-PCR of miRNA content from oncosomes isolated from the conditioned media of chemoresistant, radioresistant, and sensitive cancer cells that showed differentially expressed miRNAs as well as an exclusive set of miRNAs in each group [10,28,36,37,38]. Further experiments with transfections of selected deregulated oncosome-derived miRNA mimics and inhibitors confirmed their involvement with the development of resistance [27,39]. The most commonly highlighted mechanisms of how miRNA drive chemoresistance is apoptosis evasion by the downregulation of Bcl2 by miR-34a and miR-30a [28,37], downregulation of caspase 3/7 by miR-4443 and miR-4488 [38], and PTEN and PDCD4 by mir21 and miR-30a [10].

Table 1 presents all secretory miRNAs that were associated with the development of resistance to CT and radiotherapy (RT) of non-small cell lung cancer (NSCLC). Few studies looked at the profiles of miRNAs shuttled in oncosomes from radioresistant cancer cells, although numerous works analyzed their intracellular levels and showed that they vary for different doses and cell types, with only a few of them being shared, confirming miRNA radioresistant and sensitizing ability with transfection experiments using mimics and inhibitor vectors, demonstrating therapeutic and diagnostic potential [40].

### 2.3. Stromal Cell Secretome Mediates Resistance

To model an entire complexity of TME in vitro, it is important to consider other key players of resistance induction, such as CAFs and MSCs. CAFs are stromal tumor cells that become activated and recruited by CSCs to facilitate tumor progression. CAF exosomes (CAE) supply cancer cells with nutrients to help them grow during starvation, as well as regulatory factors to mediate response to stresses, such as hypoxia and anti-cancer therapy [58]. A growing number of studies are showing the role of CAE secretome in the chemoresistance of cancer cells, involving reduction of apoptosis, drug transporters, autophagy, and increase in proliferation [49,59,60,61,62,63]. CAE also maintain a CSC population within a tumor by wnt signaling, causing cancer cells to dedifferentiate and become stem-like [60,64,65]. In addition, CAFs form a rigid extracellular matrix with the help of collagen, integrin, and other proteins that shield the tumor and can constrict the blood supply, creating hypoxia to reduce drug penetration into the tumor [66]. CAF-derived chemokines and other factors protect cancer cells from radiation by increasing reactive oxygen species levels, DNA damage repair, and autophagy [67,68]. CAFs are radioresistant, and in response to IR, they become senescent and secrete factors that can increase the proliferation of cancer cells and promote higher viability upon irradiation [69].

MSCs are multipotent stem cells that are found in bone marrow, adipose tissue, and other organs and can travel to distant sites in the body and differentiate into osteoblasts, adipocytes, chondrocytes, or fibroblasts [70]. MSCs can also migrate to tumor sites where they communicate with the surrounding cancer cells or differentiate into CAFs. MSC secretome can protect cancer cells from anti-neoplastic therapy via oncosome-shuttled miRNA and lncRNA [71,72]. MSC communication with the tumor is very complex, and in some cases, they exert anti-cancer properties and help fight the disease [73,74]. This opened up a venue for the development of MSC exosome-based therapy to sensitize cancer cells for treatment with CT or RT.

### 2.4. Crosstalk between Radio- and Chemoresistance

Anti-cancer therapy exerts systemic effects throughout the body, causing direct tissue toxicity in the case of CT, while with RT, these effects are mediated via cellular signaling known as the bystander effect. With IR exposure, cells respond with secretome changes that become manifested in distant tissues. While the bystander effect refers to damages in unexposed cells that are connected systemically, it is the same mechanism that can also establish resistance to subsequent therapy.

CRT can be delivered concurrently or sequentially before (neoadjuvant) or after (adjuvant) the primary treatment (surgery or RT). During sequential CRT, failure can be associated with cross-resistance. Radio- and chemoresistance are two distinct phenotypes, and their interplay is very complex, in some cases offering protective effects with subsequent CT or radiation exposure, while in other cases potentiating the damaging effects. There is evidence that chemoresistant cancer cells also become resistant to ionizing radiation exposure [75,76,77,78,79,80]. In ovarian cancer cells, cross-resistance is facilitated by increased glutathione content, as glutathione inhibition with buthionine sulfoximine sensitized cisplatin-resistant cells to IR comparable to the sensitive cells [76,81]. Better survival of cisplatin-resistant glioma cells upon exposure to low-dose rate as opposed to high-dose rate suggests their increased repair capacity [78]. However, cross-resistance is not always the case and there is evidence of chemoresistant cells’ sensitivity to IR, in particular to high linear energy transfer (LET) neutrons [82,83,84]. High LET radiotherapy involves heavy particles such as fast neutrons and C-ions that are a preferrable treatment for some types of tumors with high repair capacity, such as the prostate, salivary gland tumors, osteosarcoma, and chondrosarcoma, as well as in the case of photon resistance [85]. Densely ionizing radiation makes more penetrations per unit of matter, creating damaging tracks, and it has a higher relative biological effectiveness, making a more lethal cellular impact.

In some cases, such as breast cancer, during the course of CRT, CT can be given following RT [86,87]. Research shows that radioresistant cancer cells also become resistant to CT drug exposure and can facilitate cross-resistance via oncosome secretion. A recent study found that radioresistant lung adenocarcinoma cells were less sensitive to pemetrexed treatment after long-term fractionated radiotherapy, and the authors argued that this was due to the significant downregulation of folate receptor alpha (FRα), which caused a decreased drug uptake into the cell [88]. Radioresistant nasopharyngeal carcinoma cells also show resistance to cisplatin, which is associated with an upregulation of a transmembrane transporter SLC1A6 that was confirmed with a siRNA transfection [89]. Another study also showed that radioresistant NSCLC cells had a significantly higher IC50 of cisplatin [90]. A study showed that treatment of different types of breast cancer cells with exosomes derived from radioresistant cells leads to higher viability after doxorubicin treatment [24]. On the contrary, a study [91] showed a drastic increase in the sensitivity of radioresistant A549 cells to topoisomerase 1 inhibitor SN-38 that was associated with the downregulation of the efflux transporter breast cancer resistance protein (BCRP) after long-term fractionated irradiation that resulted in increased drug accumulation inside the cell. BCRP was originally identified in breast cancer tissue but can be found in different cell types, and it is a major transporter of anti-cancer drugs out of the cell.

The main mechanism of cross-resistance development during CRT is the different modes of action of CT and RT that can lead to the formation of a more heterogenous cell population with altered gene expression and mutations that can evade death upon subsequent exposure [92]. In the case of CT with drug combinations, multiple-drug resistance (MDR) can develop due to drugs acting on the same target, wherein the first line of exposure can deregulate or cause the mutation of the target to become unresponsive [92]. MDR can also be facilitated via ABC transporter upregulation such as glycoprotein P, BCRP, and multidrug resistance-associated proteins that efficiently export drugs out of the cell. Repeated cisplatin exposure in mice leads to MDR associated with increased levels of DNA damage repair efficiency and gene expression, and it is a likely mechanism for photon RT and CT cross-resistance since DNA damage response pathways tend to be more active in resistant cells [93,94]. Further research is needed to elucidate whether the mechanisms driving cross-resistance are potentiated by CM or oncosomes.

### 2.5. Cancer Stem Cells

CSCs represent a subset of tumor cells that possess a tumor-initiating ability and can differentiate into heterogenous non-stem tumor cells [95,96]. The frequency of CSCs varies broadly between different tumor types, spanning from small populations of <1% in human acute myeloid leukemia and liver cancer up to 82% in acute lymphoblastic leukemia [96]. CSCs display cancer-type-specific CD markers on their surface that can be used for their isolation [97,98,99]. CSCs with different combinations of surface markers vary in their IR sensitivity from highly sensitive to highly resistant and thus pose an additional obstacle for cancer therapy [100]. For a successful clinical outcome, CSCs have to be completely eradicated since the remaining cells can self-renew and differentiate to entirely recover a heterogenous tumor. CSCs can be isolated using surface markers and a fluorescence-activated cell sorting or magnetic-activated cell sorting approach or with a cell sphere formation serum-free culture without attachment. Of note, only the first few passages of isolated CSC remain enriched in a cell culture, since they quickly differentiate and form a heterogenous cell population with a minor subset of CSCs. Researchers collected exosomes from CSCs, showing that they are implicated in tumor progression and mediate proliferation, hypoxia response, angiogenesis, metastasis, and therapy resistance [101].

Research is lacking about how CSC-derived oncosomes facilitate resistance, but one study showed that oncosomes isolated from gemcitabine-resistant pancreatic CSCs mediate gemcitabine resistance after incubation with sensitive BxPC cells via mir-210 upregulation and mTOR activation [102]. Mir-210 upregulation was also implicated with gemcitabine resistance in resistant non-stem pancreatic cancer cells [103]. Another study also showed that mir-155 was upregulated in oncosomes from breast CSCs as well as non-stem chemoresistant MDA-MB-231 cells and could induce doxorubicin and paclitaxel resistance of sensitive cells via epithelial mesenchymal transition induction [104]. There is evidence that transfection of pancreatic cancer cells with mir-205 mimic sensitized pancreatic gemcitabine-resistant CSCs and non-stem MIA PaCa cells to gemcitabine, and this effect was confirmed with gemcitabine administration of tumor-bearing mice generated with gemcitabine-resistant MIA PaCa cells transfected with mir-205 overexpressing lentivirus [105]. With the ability to selectively isolate and culture CSCs, more research is needed to highlight the oncosome content profile involved with radio- and chemotherapy resistance and whether it differs from non-stem-resistant cancer cells. So far, there appears to be a striking similarity in CSC and non-stem-resistant cell secretome that could be due to resistance-acquiring cells’ dedifferentiation to become stem-like. Thus, it is possible that resistant cancer cells and CSCs can be targeted by the same therapeutic approaches.

## 3. Diagnostic Biomarkers of Resistance

The detection of secretory factors holds potential as a diagnostic biomarker for tumor cell resistance status to predict a therapy response. miRNAs are a promising tool in biomarker development, as they are highly stable molecules in circulation [30]. Minimally invasive diagnostic approaches have been made using plasma levels of some miRNAs. miR-208a holds potential as a serum biomarker of NSCLC radioresistance, while other detected differentially expressed miRNAs await further investigation [39]. miR-29a-3p and miR-150-5p from blood were also found to be reflective of NSCLC radioresistance [36]. Eleven serum miRNAs were predictive of NSCLC patients’ resistance to RT [106]. Various circulatory exosome-shuttled miRNAs were also predictive of chemotherapy resistance status, as presented in a review [107]. Candidate secretory miRNAs involved with radio- and chemoresistance of NSCLC as an example are presented in Table 1, and they can be a starting point in the development of a minimally invasive diagnostic panel. Other diagnostic approaches are based on liquid and tumor biopsies, which can include miRNA from isolated oncosomes or other secretory factors, as well as intercellular miRNA analysis. Candidate intercellular miRNAs predictive of chemo- or radioresistance status have been proposed in numerous studies and are summarized in reviews [108,109].

The Human miRNA Disease Database [110] contains miRNA–disease associations, including cancer and others, from 19,280 scientific articles as of today. Cancer-associated miRNA biomarkers have been validated, and a “miRview-mets2” panel was created for the clinical identification of metastatic cancer origins, in addition to other clinical miRNA-based tests [32]. Creating a resistance status database would be helpful to current diagnostics in the clinic to select a more efficient treatment regimen.

### 3.1. Therapeutic Approaches

Since the resistance-mediating effects of oncosomes were characterized, the inhibition of their secretion, biogenesis, or uptake was attempted in combination with anticancer therapy that led to tumor sensitization and higher antineoplastic efficiency in multiple in vitro studies, as summarized in a review [107]. The administration of exosome inhibitors heparin and simvastatin can help alleviate the detrimental effects of the oncosome injection derived from resistant cells in mice [23,111]. Alkylation of TME also reduces oncosome release, and intraperitoneal injections of proton pump inhibitors in combination with chemotherapy in mice led to decreased plasma exosome levels; however, no differences in tumor weight were noted due to selected time intervals [112].

Upon the discovery of secretory regulatory RNA factors conveying chemo- and radioresistance, approaches were made to up- or downregulate them in vivo. Injections of exosomes containing mir-214 antagomir sensitized lung tumors in mice, pre-treated with oncosomes from gefitinib-resistant PC9 cells [46]. As an example, antisense oligonucleotide targeting allowed for the knockout of circITGB6 in vivo with intraperitoneal injections, and in combination with cisplatin treatment, it led to significantly lower ovarian circITGB6-transfected tumor size and increased survival in mice compared to cisplatin alone [1]. Knockdown of IL-11 in mice with lung cancer also led to better effects of cisplatin treatment [3]. Inhibition of a paracrine factor PAI-1 in mice with lung cancer by oral administration of tiplaxtitnin successfully sensitized tumors to radiation therapy and led to a significantly decreased tumor volume [5].

The animal studies discussed above provide a valuable model for the in vivo investigation of therapeutic opportunities and successful outcomes proceeding further to clinical trials. Preliminary investigations were carried out on cell cultures, while 3D cultures were also used to simulate tumor formation in vitro. Recent neo-organoid developments are a very promising treatment based on cell integration into a 3D scaffold with the following implantation into the body. Neo-organoid implantation with Matrigel-imbedded MSCs overexpressing IL-12 led to significantly better results, as compared to non-genetically modified MSCs with 67% of mice with breast cancer xenografts being completely tumor free 55 days after treatment [113].

Such approaches pave the way to abrogate the subset of resistance-acquiring cancer tumor cells via the acquisition of secreted factors. However, the question remains open as to how kill the resistant and CSCs that are also present as a subset of a heterogenous tumor in this model. Today, the major directions in CSC-targeted therapy research include immunotherapy, inhibition of key signaling pathways, inhibition of DNA repair, and awakening quiescent CSCs [114,115]. Studies of differentially expressed intracellular miRNAs between resistant and sensitive cancer cells point out the miRNA control of cancer cells’ response to IR or CT, and its direct manipulation can be used to sensitize the tumor prior to therapy. Table 2 presents a summary of the candidate deregulated miRNA of resistant NSCLC cells as an example of possible therapeutic targets.

**Figure 2:**
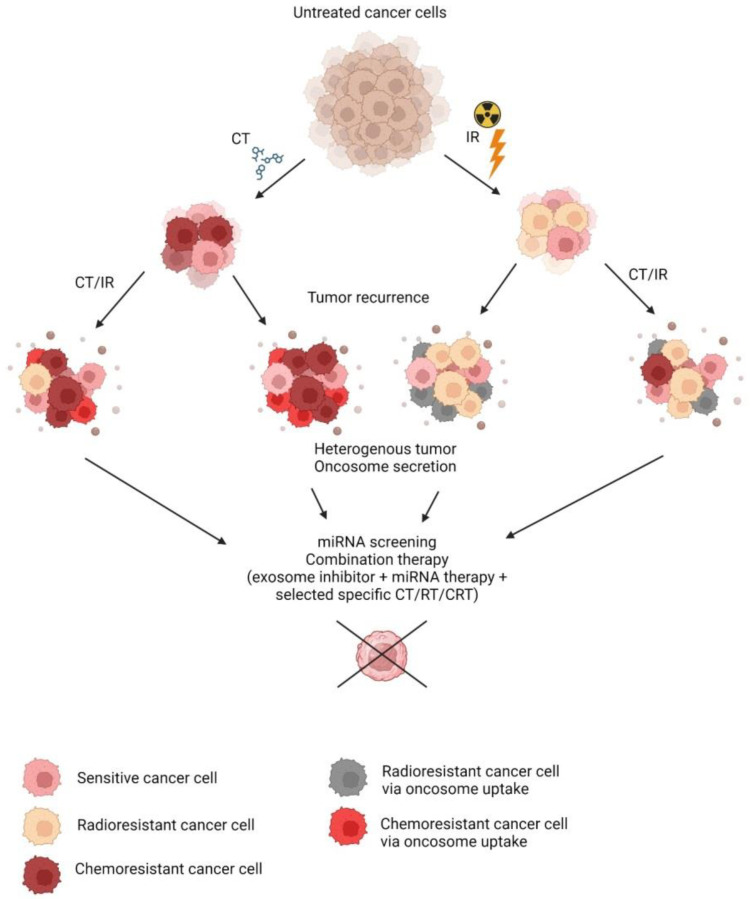
Development of tumor chemo- and radioresistance. Abbreviations: IR—ionizing radaiation; CT—chemotherapy; RT—radiotherapy; CRT—chemoradiotherapy.

miRNA therapeutic approaches for the treatment of cancer are gaining attention, with some of them in clinical trials already [171,172,173]. Several biopharmaceutical companies are developing and implementing miRNA-based therapeutics and are trying to overcome the challenges associated with tumor tissue specificity, off-target activity due to miRNA pleiotropic nature, and toxicity with novel drug delivery systems and combinations with other medications [174,175]. Targeting resistance-conferring miRNA for tumor sensitization in combination with RT or CT also has a potential to overcome resistance and provide more satisfactory therapeutic results (Figure 2). One of the ways to implement it was attempted with intra-tumoral injections of exosomes derived from MSCs transfected with selected miRNAs to sensitize cancer cells to CT, as summarized in a review [73].

Abolishment of senescent cells improves anti-cancer therapy results and can involve senolytic agents, such as chimeric antigen receptor T cells against uPAR marker and proteolysis-targeting chimera technology, in addition to other natural and targeted senolytic compounds that cause senescent cell death [176,177,178]. Senomorphic agents block SASP effects without causing senescent cell death [179]. Senotherapeutics are used as an adjuvant therapy to ablate senescent cells formed after CT or RT and lead to a better response in some patients; however, the treatment outcomes are not consistent [180].

## 4. Conclusions

The present work provided a review of action of various factors secreted from chemo- and radioresistant cancer cells, CSCs, MSCs, and CAFs that induce resistance in unexposed cancer cells. This is one of the mechanisms associated with disease recurrence, and this knowledge is important in paving the way to abolish the development of resistance upon previous RT or CT. While there is a big step forward in elucidating the secreted factors in the CM of chemoresistant cancer cells that facilitate the development of resistance, more research is needed to uncover such factors and their mechanisms of action for radioresistant cancer cells. A deeper understanding is also required for cross-resistance development via secreted factors as it has implications in CRT planning. Overcoming resistance will lead to more consistent treatment outcomes. Future clinical studies are needed to validate secretory miRNAs as biomarkers to diagnose the chemo- and radioresistance status of oncology patients. The high heterogeneity of tumors, even of the same types, between patients calls for the approaches of personalized medicine. However, the development of a strong diagnostic base from existing cases will aid in the selection of a therapeutic strategy for a particular patient and contribute to the search for a comprehensive approach in overcoming resistance.

## Figures and Tables

**Table 1:** Secretory miRNA (exosomal and circulatory) involved in the radio- and chemoresistance of NSCLC.

Type of Resistance	miRNA
Tyrosine kinase inhibitors	mir-BART14, mir-1469, mir-16-1, mir-196, mir-4791, mir-4796, mir-548aq, mir-72, mir-H19, mir-138-2, mir-153, mir-585, mir-4803, mir-744, mir-769 [41] mir-184, mir-3913 [42] mir-658, mir-564 [43] mir-1468, mir-23 [44] mir-136 [45] mir-214 [46] mir-210 [47] mir-615 [48]
Cisplatin	mir-20a [49] mir-193a [50] mir-524 [51] mir-4443 [52] mir-1246 [53] mir-425 [54] mir-103a [55] mir-1273a [56] mir-100 [27]
IR	mir-196a [57] mir-208a [39] mir-29a, mir-150 [36]

**Table 2:** Most commonly differentially expressed miRNA of radio- and chemoresistant NSCLC cells. IR—ionizing radiation; EGFR-TKI—epidermal growth factor receptor tyrosine kinase inhibitors; 5FU—5-fluorouracil.

miRNA	Number of References	Type of Resistance
mir-21	13	IR [116,117] Cisplatin [118,119,120] EGFR-TKI [121,122,123,124] 5FU [125] Cisplatin and paclitaxel [126] Cisplatin and docetaxel [127] Cisplatin, docetaxel, and IR [128]
mir-145	10	IR [129] Cisplatin [130,131] EGFR-TKI [132,133,134] Docetaxel [135] Pemetrexed [136] Paclitaxel [137] Cisplatin and pemetrexed [138]
mir-200c	6	ALK-TKI [139,140] EGFR-TKI [141,142] Paclitaxel [143] Vincristine, cisplatin, and MDR [144]
mir-17	6	Cisplatin [145,146] Paclitaxel [147,148] EGFR-TKI [149,150]
mir-34a	5	Cisplatin [151,152,153] Gefitinib [154,155]
mir-326	5	Cisplatin [156,157,158] Matrine [159] Gefitinib [160]
mir-200a	5	Cisplatin [161,162] TKI [163,164,165]
mir-200b	5	Docetaxel [166,167,168,169,170]

## Data Availability

Not applicable.
